# Impact of Orthognathic Surgery on Quality of Life in Patients with Dentofacial Deformities

**DOI:** 10.1155/2017/4103905

**Published:** 2017-09-27

**Authors:** Faezeh Eslamipour, Arash Najimi, Azade Tadayonfard, Zeinab Azamian

**Affiliations:** ^1^Torabinejad Dental Research Center, Department of Oral Public Health, School of Dentistry, Isfahan University of Medical Sciences, Isfahan, Iran; ^2^Department of Medical Education, Medical Education Research Center, Isfahan University of Medical Sciences, Isfahan, Iran; ^3^Department of Prosthodontics, School of Dentistry, Shahid Beheshti University of Medical Sciences, Tehran, Iran; ^4^Department of Orthodontics, School of Dentistry, Isfahan University of Medical Sciences, Isfahan, Iran

## Abstract

The aim of this investigation was to determine the impact of orthognathic surgery on quality of life in patients with dentofacial deformities at immediate presurgery and at 3-week, 3-month, and 6-month intervals following the surgery. Subjects included forty-three 18–40-year-old Iranian orthognathic patients who were referred to private offices in Isfahan. Data collection was performed using the 22-item Orthognathic Quality of Life Questionnaire (OQLQ). Participants completed the questionnaire prior to surgery and 3 weeks, 3 months, and 6 months after it. Differences and correlations were calculated by the two-tailed *t*-test, ANOVA with Repeated Measure test, and the Pearson correlation coefficient. The results showed significant reduction returned to baseline in OQLQ mean scores and aesthetic, awareness, and social subdomains in all 3 intervals after surgery. However oral function domain showed an increase at *T*_2_ and then a decrease at next intervals. Maximum and minimum effect size were observed in aesthetic (ES = 0.7) and oral function (ES = 0.3) domain, respectively. Based on the finding of this study, in 6-month interval after surgery, orthognathic surgery causes significant improvements in quality of life in patients with dentofacial deformities as assessed in emotional, psychological, oral function, and social domains and maximum changes occurred in emotional domain.

## 1. Introduction

Dentofacial deformities have been described as changes that primarily affect the jaws and teeth, although the multiple craniofacial structures may also be affected [1]. In most cases, they are the result of moderate or severe genetic distortions of the normal development process (such as mandibular prognathism, bimaxillary prognathism or retrognathism, maxillary vertical excess) and should be corrected using an integrated treatment of orthodontics and orthognathic surgery in adult orthodontic [2]. Some study reported these deformities affect 20% of the population with various degrees of functional and esthetic compromise [3]. In a study, Proffit and White revealed that a large part of the population in the USA suffer from significant malocclusion. In many of these cases, the facial proportions were abnormal and approximately 5% were so severe that the patients could be regarded as handicapped [4]. Borzabadi-Farahani et al. reported that 36% of Iranian young population need orthodontic treatment and nearly 12% of them had severe malocclusion which needs compound orthodontics and orthognathic surgery treatments [5, 6].

One of the important risk factors in low appearance self-esteem is determined to be severe dentofacial deformity [7]. Over the years, the patients with dentofacial deformities have lower mean quality of life values in comparison with those who have not [8]. At present, the combination of the two treatment modalities, maxillofacial surgery and orthodontics, is one of the most important parts in the corrective treatment of malocclusion and facial deformities [9]. Current advances in diagnostic and planning methods and surgical techniques have made orthognathic surgery safe and common for treating these deformities [10]. Modern fixation techniques (i.e., internal rigid fixation) and improvements in facial esthetics have increased patients' trust in this type of surgery, resulting in an increased demand for orthognathic procedures [11].

Patients' motivation for the surgical option is the hope for improvement of their quality of life [12]. The information about the effect of orthognathic surgery on patients during the recovery phase enables surgeons to better inform patients on their expectations from surgery [13].

Al-Bitar et al. in a study revealed that Jordanian patients with dentofacial deformities as an Arab population had generally lower score and therefore a poorer QOL than reported in British, Japanese, and Chinese populations. They concluded that these differences may be cultural or may be due to differences in healthcare system's criteria for funding. The differences may refer to socially and culturally unique definitions and concepts of health and quality of life and awareness of higher level needs [13]. Due to paucity of published studies investigating QOL in Iranian orthognathic patients, the aim of this study was to determine changes in QOL among Iranian patients with dentofacial deformities who had undergone orthognathic surgery after completion of presurgical orthodontic treatment for dental decompensation.

## 2. Methods

### 2.1. Participants

This prospective study was carried out on sixty 18–40-year-old orthognathic patients who had completed presurgical orthodontic phase in the orthodontists private offices in Isfahan, Iran, and were scheduled to undergo orthognathic surgery. A convenient sampling was used. All the subjects mainly had sagittal and/or vertical discrepancy or asymmetric jaw relation and underwent bimaxillary osteotomies. Exclusion criteria were patients with cleft lip and palate or craniofacial syndromes, patients who were scheduled to undergo orthognathic surgery without orthodontic treatment or with additional features, e.g., genioplasty or distraction device, and patients reluctant to participate in the study. The study trend was approved by the research committee, Faculty of dentistry, Isfahan University of Medical Sciences, Iran (no. 388488). The research protocol was described to patients and a written consent was obtained before participating in the study. This study has been conducted in full accordance with the World Medical Association Declaration of Helsinki.

### 2.2. Instruments and Data Collection

Data were collected using self-administered 22-item Orthognathic Quality of Life Questionnaire (OQLQ a condition-specific QOL assessment). The Orthognathic Quality of Life Questionnaire (OQLQ) was developed as an instrument to estimate quality of life in patients treated with orthognathic surgery in 2000 and validated in 2002 by Cunningham et al. [14, 15].

The questionnaire was translated into Persian using a standardized forward-backward linguistic translation method. The content validity of the questionnaire was approved by orthodontic and psychiatric specialists. In a pilot study the face validity was checked and the internal consistency reliability was assessed by measuring Cronbach's alpha (*α* = 0.93).

The OQLQ questionnaire consists of 22 questions which is rated on a 5-point Likert scale ranging from “does not bother me at all” (score 0) to “bothers me a lot” (score 4). The total score is 0 to 88. A lower score indicates a better QOL and vice versa. This questionnaire consists of 4 subscales: aesthetic impact (items 1, 7, 10, 11, and 14, range 0 to 20), oral function (2 to 6, range 0 to 20), awareness impact (8, 9, 12, and 13, range 0 to 16), and social impact (items 15 to 22, range 0 to 32).

The questionnaire was distributed to the patients and asked them to complete it during their routine visits in orthodontist offices at 4 times (*T*_1_–*T*_4_), first, the last visit before surgery (about 10–20 days before surgery), second, in 3 weeks after surgery, third, in 3 months after surgery, and the last, 6 months after surgery. Before completing the questionnaire by patients, instructions were given regarding the study.

In the copy of the last questionnaire, which the patients filled 6 months after surgery, global measures were included to rate patient satisfaction with two questions as follows: “How would you rate your satisfaction with the outcome? Rate your satisfaction with a score from 0 to 5 where 0 means completely dissatisfied and 5 means completely satisfied.” The other question was “would you recommend this surgery to other people?” with a “yes” or “no” answer. The average time for a patient to fill the questionnaire ranged from 5 to 7 minutes.

### 2.3. Data Analysis

Data were analyzed using the statistical software SPSS version 18 (Inc., Chicago, IL) The Kolmogorov Smirnov normality test was performed to determine if the samples conformed to a normal distribution. Differences between subgroups and correlations between QOL as dependent variables and age, sex, skeletal problem, and level of their education as independent variables were calculated by the two-tailed *t*-test. Regarding nonnormal distribution of OQLQ and its subdomains scores, Friedman and Wilcoxon analysis were performed. The magnitude of the statistical difference in scores was determined by calculating effect sizes (ES); ES (effect size) was calculated with division of mean changes by standard deviation for evaluating the efficacy of orthognathic surgery in terms of clinical importance on patient's quality of life. An ES of less than 0.2 is considered as minimal; 0.2 to 0.49 as small; 0.5 to 0.8 as moderate; and greater than 0.8 as large. The larger the ES, the greater the magnitude of change as a result of the intervention. A *P* < 0.05 was considered significant. Reliability analyses were conducted to assess the consistency by calculating Cronbach's alpha. To be evaluated as a reliable scale, an *α* of at least 0.70 was required.

## 3. Results

From sixty patients who agreed to participate in the study only 43 patients had completed the questionnaires at all-time points, which were used in the final analyses so the response rate was 71.6%. Demographic characteristics of patients are presented in Table 1.

**Figure 1:**
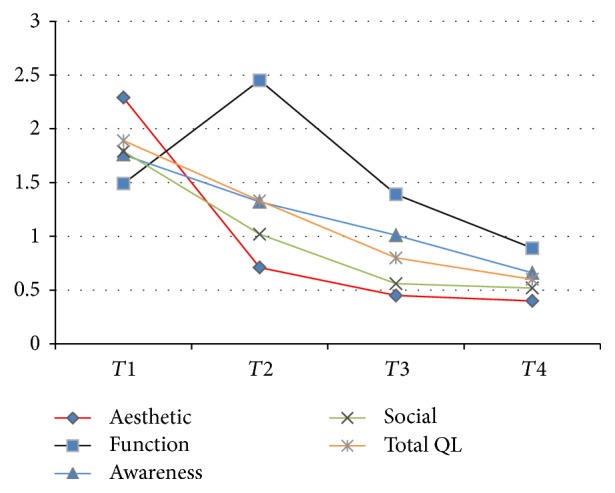
Mean of total OQLQ and subdomains (range 0–4) in different stage of treatment.

Mean change values of OQLQ and its subdomains scores in different stage of treatment are showed in Table 2. A significant reduction in OQLQ and all subdomains mean scores was observed over the trajectory of treatment (Figure 1).

There was not any statistically significant relation between OQLQ mean scores and demographic characteristics of samples except gender at presurgical stage *T*_1_ and type of dentofacial problem at 6-month postsurgical stage *T*_4_ (Table 3).

Based on our results effect size value of change in OQLQ was 0.63. Throughout the treatment period, variation in subdomain scores differed significantly compared with the baseline. Maximum and minimum ES were observed in aesthetic (ES = 0.7, *P* < 0.001) and oral function (ES = 0.3, *P* = 0.001) domain, respectively. An exception was seen in oral function domain over the time of assessment. It showed an increase at *T*_2_ and then a decrease at *T*_3_ and *T*_4_ (Table 4).

At 6 months, 81.4% of patients reported their global satisfaction above 3 (score 0–5) and the mean score of it was 4.07 ± 1.18 and 79.1% claimed they would recommend the surgery to others.

## 4. Discussion

Patients with dentofacial deformities, who visit a clinic for the first time, frequently behave in a shy, defensive, and passive manner because of a lack of confidence in their appearance [12, 16]. Their appearance leads to impacts and influences on many aspects of life such as social interactions, chances when seeking employment, being chosen as a partner, and their personality characteristics; therefore it affects their QOL. In these patients orthognathic surgery has become more important because it has been widely performed to improve dentofacial deformities [17, 18].

This study showed that there were significant improvements in patients' quality of life both at 3 and 6 months after orthognathic surgery compared with baseline levels. This finding is consistent with similar studies conducted in China and USA using the same questionnaire [9, 19]. Moreover, this finding is supported by many other studies that show remarkable improvements in patients' wellbeing in different aspects including psychological, functional, social, emotional, and physical wellbeing [4, 7, 10, 15, 20–23]. These findings illustrate the effectiveness of orthognathic surgery beyond its complications like swelling, pain, and so forth.

In this study, at 6 months there was a significant improvement in QOL compared with 3 months' interval. This gradual postsurgical improvement is supported by Choi et al.'s study which reported moderate to large improvement in this interval [9].

In terms of different aspects of patients' QOL, maximum changes occurred in emotional domain and then social, psychological, and functional aspects, respectively. These results are similar to previous studies [3, 4, 9]. Based on our results minimum changes occurred in the functional domain and no significant change occurred as early as 3 months. This is also similar to the results of Choi et al. which revealed no significant or even some reduction in patient's short-term wellbeing after the surgery [9]. Desforges et al. showed that improvement in this domain occurred later than other domains [22]. This finding is expected, because surgical intervention is not without complication in itself. Pain, swelling, neurosensory disturbances, limitation in mouth opening, and reduced muscular efficiency are common morbidities [19, 20, 24, 25].

Previous studies comparing the total score of patients in pre- and postsurgical periods suggested that changes in self-esteem and self-confidence had occurred [4, 26, 27]. Some investigations reported that patients had received more psychological benefits such as improvement of body image, facial image [28], and higher interpersonal relationship [27].

The results indicate short-term effects of orthognathic surgery are more remarkable and rapid on emotional and social wellbeing rather than physical status. This can be a reflection of orthodontic treatment phase and also the patient's dental and neuromuscular adaptation. In terms of improvement period, minimum period was seen in emotional domain in the same early stages after the surgery [9, 29].

According to this study, women's overall QOL score in all four domains (specially, in emotional and social subscales) showed a poorer status compared with men before surgery. However, women's QOL achieved a remarkable improvement in all 4 aspects in the same range with men after the surgery. This indicates that female QOL had more improvement. These findings offer support for some studies [21, 25]. Siow et al. suggested that improvement of self-confidence after surgery in women is more than men [30]. Nicodemo et al. using SF-36 reported higher mean score in emotional domain in female patient [31]. In contrast Choi et al. did not find association between gender and QOL [9]. This may be due to cultural differences.

Pahkala and Kellokoski suggested that patients who had mandibular setback were more satisfied than patients with skeletal class II that had mandibular advancement [32]. Our findings are in agreement with that study. In contrast Choi et al. showed that there was no significant difference in QOL scores between different dentofacial deformities in any time point after surgery [9]. More studies are necessary to clarify the relation between malocclusion type and changes QOL after orthognathic surgery.

According to results of previous studies patients undergoing surgery for dentofacial deformity are mostly satisfied with the surgical results [4, 19, 33–35]. Profit stated that almost all the patients that had gone under surgical procedures reported long term satisfaction (80–90% depend on deformity types). The same number of patients stated that according to their experience of surgery they have suggested others to such treatment and have been ready to experience it once again [19, 36]. The same results have been shown in the present study; patients reported high levels of satisfaction and improvement regarding surgical outcome and suggest the surgery to other people. Therefore, in aggregate, orthognathic surgery in patients with dentofacial deformity is considered beneficial from the patient's point of view. Smith and Cuningham evaluated willingness-to-pay for orthognathic surgery and came to the conclusion that patients with dentofacial deformity are willing to spend more than normal control and consider surgery as a good option in terms of cost-benefit. However a cost-benefit analysis study suggested comparing benefits and costs of orthognathic surgery in patients with dentofacial deformity [37].

This study has several limitations. More patients with longer follow-up and a survey before the institution of orthodontics would be useful to better correlate the results with lasting benefits obtained through orthognathic surgery.

In conclusion, orthognathic surgery causes significant improvement in patients' quality of life in Iranian patients. This improvement was seen in emotional, oral function, psychological, and social domains of quality of life. The maximum influence was in emotional and the least in functional aspect. Our study assessed quality of life in a limited period postoperatively and obviously there is a need for longitudinal studies in this area of healthcare services.

## Figures and Tables

**Table 1:** Demographic characteristics of patients.

Variables	*N*	(%)
Sex		
Male	13	30.2
Female	30	69.8
Age group		
≤25	31	72.1
>25	12	27.9
Problem		
Class II	13	30.2
Class III	30	69.8
Education		
≤Diploma	13	31.7
>Diploma	28	69.3

**Table 2:** Mean values of OQLQ and its subdomains scores in different stage of treatment.

Variables	*T*_1_	*T*_2_	*T*_3_	*T*_4_
	Mean (SD)	Mean (SD)	Mean (SD)	Mean (SD)
OQLQ (0–88)	40.5 (21.0)	29.2 (18.5)	17.6 (13.0)	13.1 (11.0)
Aesthetic (0–20)	11.4 (6.0)	3.6 (4.3)	2.3 (3.2)	2.0(2.9)
Function (0–20)	7.4 (5.1)	12.2 (5.5)	6.7 (4.8)	4.4(4.1)
Awareness (0–16)	7.0 (4.5)	5.1 (4.7)	4.0 (4.1)	2.5 (2.9)
Social (0–32)	14.5 (9.6)	8.1 (8.5)	4.5 (4.5)	4.1 (4.0)

**Table 3:** Mean OQLQ scores of the sample based on demographic characteristics and phase of treatment.

OQLQ	*T*_1_	*T*_2_	*T*_3_	*T*_4_
	Mean ± SD	Mean ± SD	Mean ± SD	Mean ± SD
Sex				
Male	30.1 ± 20.9	24.0 ± 11.8	20.1 ± 16.0	10.0 ± 9.1
Female	45.0 ± 19.7	31.7 ± 20.7	16.5 ± 11.7	14.5 ± 11.6
*P* value	<0.03^*∗*^	0.2	0.42	0.23
Age group				
≤25	37.7 ± 19.7	28.8 ± 18.6	19.0 ± 14.4	13.9 ± 12.2
>25	47.8 ± 23.3	30.1 ± 19.2	13.7 ± 17.6	11.2 ± 7.0
*P* value	0.16	0.86	0.25	0.48
Problem				
Class II	43.7 ± 19.8	31.9 ± 23.9	21.3 ± 11.1	19.1 ± 12.1
Class III	39.1 ± 21.7	28.0 ± 16.1	16.0 ± 13.6	10.5 ± 9.6
*P* value	0.51	0.57	0.24	0.018^*∗*^
Education				
≤Diploma	38.8 ± 23.0	25.9 ± 19.0	16.7 ± 12.8	14.1 ± 14.4
>Diploma	40.5 ± 20.0	30.6 ± 18.5	18.1 ± 13.6	13.4 ± 9.4
*P* value	0.75	0.48	0.76	0.84

^*∗*^ *P* < 0.05 is significant.

**Table 4:** Comparison of OQLQ and its subdomains from baseline (*T*1) to postoperative 6+ months (*T*4).

Variables	*T*_1_-*T*_2_			*T*_1_-*T*_3_			*T*_1_-*T*_4_			*T*_2_-*T*_3_			*T*_2_-*T*_4_			*T*_3_-*T*_4_		
	Mean	*P* ^*∗*^	ES^*∗∗*^	Mean	*P* ^*∗*^	ES^*∗∗*^	Mean	*P* ^*∗*^	ES^*∗∗*^	Mean	*P* ^*∗*^	ES^*∗∗*^	Mean	*P* ^*∗*^	ES^*∗∗*^	Mean	*P* ^*∗*^	ES^*∗∗*^
	(SD)			(SD)			(SD)			(SD)			(SD)			(SD)		
OQLQ	9.27	0.02	.22	21.95	<0.001	.54	27.37	<0.001	.63	10.85	0.001	.32	16.35	<0.001	.47	5.02	<0.001	.20
	(23.2)			(23.9)			(22.0)			(17.2)			(14.2)			(8.3)		
Aesthetic	7.64	<0.001	.59	8.95	<0.001	.67	9.48	<0.001	.70	1	0.219	.12	1.83	0.001	.25	.62	0.163	.10
	(7.3)			(7.0)			(6.3)			(4.7)			(2.9)			(2.7)		
Function	−5.43	<0.001	−.45	0.25	0.79	.02	3.02	0.001	.30	5.05	<0.001	.42	7.83	<0.001	.62	2.35	<0.001	.25
	(5.4)			(6)			(5.5)			(5.8)			(6.1)			(3.2)		
Awareness	1.48	0.018	.15	2.95	0.001	.33	4.46	<0.001	.50	1.08	0.102	.12	2.43	0.001	.29	1.47	<0.001	.20
	(3.6)			(5.1)			(4.9)			(3.8)			(4.1)			(2.7)		
Social	5.56	0.008	.28	9.8	<0.001	.55	10.39	<0.001	.57	3.71	0.006	.26	4.2	<0.001	.30	.57	0.335	.06
	(1.9)			(10.9)			(9.8)			(7.5)			(4)			(3.7)		

^*∗*^ *P* < 0.05 is significant; ^*∗∗*^ES = effect size: <0.2 = minimal change; 0.2–0.49 = small change; 0.5–0.8 = moderate change; >0.8 = large change.
